# Dysregulation of microRNA expression drives aberrant DNA hypermethylation in basal-like breast cancer

**DOI:** 10.3892/ijo.2013.2197

**Published:** 2013-11-29

**Authors:** RUPNINDER SANDHU, ASHLEY G. RIVENBARK, RANDI M. MACKLER, CHAD A. LIVASY, WILLIAM B. COLEMAN

**Affiliations:** 1 Department of Pathology and Laboratory Medicine, University of North Carolina School of Medicine, Chapel Hill, NC 27599, USA; 2 UNC Program in Translational Medicine, University of North Carolina School of Medicine, Chapel Hill, NC 27599, USA; 3 UNC Lineberger Comprehensive Cancer Center, University of North Carolina School of Medicine, Chapel Hill, NC 27599, USA

**Keywords:** microRNA, aberrant DNA hypermethylation, primary breast cancers, epigenetic silencing, basal-like breast cancers

## Abstract

Basal-like breast cancers frequently express aberrant DNA hypermethylation associated with concurrent silencing of specific genes secondary to DNMT3b overexpression and DNMT hyperactivity. *DNMT3b* is known to be post-transcriptionally regulated by microRNAs. The objective of the current study was to determine the role of microRNA dysregulation in the molecular mechanism governing DNMT3b overexpression in primary breast cancers that express aberrant DNA hypermethylation. The expression of microRNAs (miRs) that regulate (miR-29a, miR-29b, miR-29c, miR-148a and miR-148b) or are predicted to regulate *DNMT3b* (miR-26a, miR-26b, miR-203 and miR-222) were evaluated among 70 primary breast cancers (36 luminal A-like, 13 luminal B-like, 5 HER2-enriched, 16 basal-like) and 18 normal mammoplasty tissues. Significantly reduced expression of miR-29c distinguished basal-like breast cancers from other breast cancer molecular subtypes. The expression of aberrant DNA hypermethylation was determined in a subset of 33 breast cancers (6 luminal A-like, 6 luminal B-like, 5 HER2-enriched and 16 basal-like) through examination of methylation-sensitive biomarker gene expression (*CEACAM6*, *CDH1*, *CST6*, *ESR1*, *GNA11*, *MUC1*, *MYB*, *TFF3* and *SCNN1A*), 11/33 (33%) cancers exhibited aberrant DNA hypermethylation including 9/16 (56%) basal-like cancers, but only 2/17 (12%) non-basal-like cancers (luminal A-like, n=1; HER2-enriched, n=1). Breast cancers with aberrant DNA hypermethylation express diminished levels of miR-29a, miR-29b, miR-26a, miR-26b, miR-148a and miR-148b compared to cancers lacking aberrant DNA hypermethylation. A total of 7/9 (78%) basal-like breast cancers with aberrant DNA hypermethylation exhibit diminished levels of ≥6 regulatory miRs. The results show that i) reduced expression of miR-29c is characteristic of basal-like breast cancers, ii) miR and methylation-sensitive gene expression patterns identify two subsets of basal-like breast cancers, and iii) the subset of basal-like breast cancers with reduced expression of multiple regulatory miRs express aberrant DNA hypermethylation. Together, these findings strongly suggest that the molecular mechanism governing the DNMT3b-mediated aberrant DNA hypermethylation in primary breast cancer involves the loss of post-transcriptional regulation of *DNMT3b* by regulatory miRs.

## Introduction

Neoplastic transformation is associated with alterations in DNA methylation, including both global hypomethylation and gene-specific hypermethylation (1–3). Hypomethylation may result in aberrant or inappropriate expression of genes that contribute to neoplastic transformation, tumorigenesis or cancer progression (oncogenes) (4). In addition, genome-wide loss of methylation contributes to chromosomal instability by destabilizing pericentromeric regions of certain chromosomes (5–7). Gene-specific hypermethylation typically reflects hypermethylation of CpG-rich regions within gene promoter sequences that lead to gene silencing events (1). Methylation-dependent gene silencing is a mutation-independent mechanism for inactivation of tumor suppressor genes (and other negative mediators of neoplastic development) in cancer (8). Methylation-dependent gene silencing is a common epigenetic modification present in breast cancer cells contributing to the initiation, development and progression of breast cancer (9–11). Recently, we identified a subset of breast cancer cell lines and primary breast cancers that exhibit aberrant DNA hypermethylation that results in concurrent epigenetic silencing of multiple methylation-sensitive genes secondary to DNA methyltransferase enzyme hyperactivity associated with overexpression of DNMT3b (12). Breast cancers that exhibit this aberrant DNA hypermethylation are substantially enriched for the basal-like molecular subtype (12). The close association of aberrant DNA hypermethylation with the basal-like molecular subtype of breast cancer strongly suggests that dysfunction of the epigenome represents a fundamental biological property that contributes to the clinical behavior of this form of breast cancer.

While the expression of the DNMT3b-related aberrant DNA hypermethylation among basal-like breast cancers is now well established, the molecular mechanism governing the hyper methylation defect has not been examined in primary breast cancer. Several studies in the literature have shown that DNMT3b is often overexpressed in different types of cancers including breast cancer (12–16). However, unlike other genes that are overexpressed in cancer as a result of genetic mutations and/or gene amplifications, the mechanisms accounting for overexpression of DNMT3b does not involve these changes (17). Likewise, inappropriate or increased trans-activation does not account for the overexpression of DNMT3b in cancer (17). Numerous studies have now demonstrated that *DNMT3b* is negatively post-transcriptionally regulated by microRNAs (miRs), which are small endogenous non-coding RNAs (19–25 nucleotide long) that have emerged as key players in regulation of gene expression (18). Post-transcriptional regulation of gene expression by miRs occurs through sequence-specific targeting of mRNAs as a result of recognition of complementary sites, most often in the 3’-untranslated region (UTR) of the target mRNA, producing either translational repression or degradation of the target mRNA (19–23). miRs are expressed in a tissue-specific manner and have been implicated in the regulation of myriad of biological processes, including cellular proliferation, differentiation, apoptosis and development (24–27). Altered miR expression is associated with several types of human cancer, including breast cancer (28–31). The dysregulated pattern of expression of miRs between normal and cancerous tissues in breast cancer has been extensively studied. The expression patterns of different miRs have been correlated with tumor stage, estrogen and progesterone receptor expression, proliferation index, vascular invasion, epithelial to mesenchymal transition, metastasis and neovascularization (29,32–34). Studies have shown that *DNMT3a* and *DNMT3b* are directly targeted by members of the miR-29 family (miR-29a, miR-29b and miR-29c) in lung cancer (35) and acute myeloid leukemia (36). Similarly, *DNMT3b* is regulated by the miR-148 family (miR-148a and miR-148b) in cell lines of multiple origin, including the MCF-7 breast cancer cell line (37). In recent investigations, we found that the mechanism accounting for overexpression of DNMT3b among breast cancer cell lines that express aberrant DNA hypermethylation is related to concurrent loss of microRNAs (miRs) that post-transcriptionally regulate *DNMT3b* mRNA, including miR-29c, miR-148a, miR-148b, miR-26a, miR-26b and miR-203 (38).

In the current study, we investigated loss of miR-mediated post-transcriptional regulation of *DNMT3b* in primary invasive breast cancers as a molecular mechanism governing the overexpression of DNMT3b that drives aberrant DNA hypermethylation in basal-like breast cancer. We analyzed 70 paraffin-embedded human primary invasive breast cancers (36 luminal A-like, 13 luminal B-like, 5 HER2-enriched and 16 basal-like) and 18 normal mammoplasty tissues for differential expression of regulatory miRs. The results show that i) significantly reduced expression of miR-29c distinguishes basal-like breast cancers from the other breast cancer clinical subtypes; ii) miR expression patterns revealed two groups among the basal-like breast cancers corresponding to those with diminished expression and those expressing normal levels of regulatory miRs; iii) loss of combinations of miR-29a, miR-29b, miR-148a, miR-148b, miR-26a and miR-26b is associated with expression of aberrant DNA hypermethylation among primary invasive breast cancers; and iv) basal-like breast cancers that exhibit aberrant DNA hypermethylation express diminished levels of miRs that post-transcriptionally regulate *DNMT3b*.

## Materials and methods

### Primary breast cancers and normal mammoplasty tissues

A total of 70 paraffin-embedded human primary breast tumors and 18 normal mammoplasty tissues were obtained from the paraffin archives of the UNC Lineberger Comprehensive Cancer Center at the University of North Carolina, School of Medicine (Chapel Hill, NC, USA). Clinical classification of primary breast cancers was accomplished by immunohistochemistry for ER, PR, HER2, CK5/6 and EGFR (39–41). The cohort of primary breast cancers evaluated included examples from each of the intrinsic molecular subtypes based upon their immunohistochemical surrogate: 36 luminal A-like (ER^+^/PR^+^/HER2^−^), 13 luminal B-like (ER^+^/PR^+^/HER2^+^), 5 HER2-enriched (ER^−^/PR^−^/HER2^+^), and 16 basal-like (ER^−^/PR^−^/HER2^−^ plus CK5/6^+^ or EGFR^+^) (42–44). Protection of patient privacy and handling of specimens followed strict policies of the Institutional Review Board of the University of North Carolina School of Medicine. The current study was reviewed by the Institutional Review Board of the University of North Carolina School of Medicine and was formally declared exempt based upon the use of existing data and existing tissue specimens that were stripped of all identifying information. Hence, patient consent was not required and was not sought.

### RNA isolation from paraffin-embedded tissues

The paraffin blocks were blinded (in terms of clinical subtypes) before selection for analysis and up to 35 mg of tissue core samples were obtained from the Translational Pathology Laboratory core facility (Department of Pathology and Laboratory Medicine, University of North Carolina School of Medicine). H&E stained sections from each paraffin block were evaluated to select areas of the blocks to be cored. This selection ensured that the cores consisted of cancer tissue or normal breast epithelium (and not stroma/fat) from primary breast cancer and reduction mammoplasty tissues, respectively. Total RNA was isolated from breast cancers and normal breast epithelium using Recover All™ Total Nucleic Acid Isolation Kit for FFPE according to the manufacturer’s instructions (cat no. AM1975, Ambion/Life Technologies, Carlsbad, CA, USA). Tissue cores were crushed and ground in liquid nitrogen, and then deparaffinized using a series of washes with Slide Brite (part no. SBT G1, Biocare Medical, Concord, CA, USA) and ethanol. Nucleic acid samples were purified using the Qiagen RNeasy mini kit (cat no. 74104, Qiagen, Valencia, CA, USA). Isolated RNA was quantified after extraction using a Nanodrop 2000 Spectrophotometer (NanoDrop Technologies, Wilmington, DE, USA).

### MicroRNA expression analysis

Members of miR-29 (miR-29a, miR-29b and miR-29c) and miR-148 (miR-148a and miR-148b) family were selected for examination based upon available literature linking them to direct post-transcriptional regulation of *DNMT3b* in lung cancer (35), acute myeloid leukemia (36) and cell lines of multiple origin (37). In addition, candidate miR regulators of *DNMT3b* were identified (38) using the computational tools of target prediction programs and resources from publicly available databases, including Miranda (http://www.microRNA.org/), TargetScan (http://www.targetscan.org/vert_42/), miRGen (http://www.diana.pcbi.upenn.edu/miRGen/v3/miRGen.html), PicTar (http://pictar.mdc-berlin.de/), and miRBase (http://microrna.sanger.ac.uk/sequences/). Target predictions were made using Gene symbol DNMT3b (Entrez Gene ID 1789 and Ensembl Gene ID ENSG00000088305). Based on high stringency *in silico* selection criteria that included PicTar score (indicative of HMM maximum likelihood fit), highly conserved miRs, and good mirSVR scores (indicative of seed-site pairing, site context, free-energy and conservation), we identified miRs that potentially target *DNMT3b* (38). The candidate miRs were prioritized based on the available literature and/or their recognition as potential candidates by multiple target prediction programs. miRs that were differentially expressed among breast cancer cells in primary cancers (29) and cell lines (45) were considered for further analysis. Based upon this computational analysis, nine miRs were selected for examination: miR-29a, miR-29b, miR-29c, miR-148a, miR-148b, miR-26a, miR-26b, miR-203 and miR-222.

miR expression analysis was accomplished by real-time PCR utilizing an ABI 7500 Real Time PCR System (Applied Biosystems/Life Technologies, Carlsbad, CA, USA) according to TaqMan miRNA assay protocol (Applied Biosystems). TaqMan MiRNA Reverse Transcription Kit (part no. 4366596, Applied Biosystems/Life Technologies) was employed to reverse transcribe the total RNA samples (10 ng) using the TaqMan miRNA specific primers (Applied Biosystems/Life Technologies) according to the manufacturer’s protocol. Real-time primers and probes for miR-29a (assay ID 000412), miR-29b (assay ID 000413), miR-29c (assay ID 000415), miR-148a (assay ID 000470), miR-148b (assay ID 000471), miR-26a (assay ID 000405), miR-26b (assay ID 000407), miR-203 (assay ID 000507), miR-222 (assay ID 002276) and RNU66 (assay ID 001002) were purchased from Applied Biosystems/Life Technologies. These assays specifically detect mature miRNAs (not pre-miRNAs). All real-time PCR reactions were performed in triplicate using TaqMan Universal PCR Master mix (cat no. 4324018, Applied Biosystems/Life Technologies) in 20 *μ*l volume containing 10 *μ*l TaqMan Universal PCR Master mix, 1 *μ*l of primers and probe mix of the miR-specific TaqMan MicroRNA Assay (Applied Biosystems/Life Technologies), 1.33 *μ*l of RT product, and 7.67 *μ*l of nuclease free water and the following amplification conditions: 95°C for 10 min, 40 cycles of 95°C for 15 sec and 60°C for 1 min. Relative expression levels for each miR were calculated based upon the expression of RNU66 and differences in gene expression were determined relative to normal breast tissues from reduction mammoplasties using the comparative Ct method described in the ABI Prism 7700 User Bulletin #2 (Applied Biosystems/Life Technologies).

### Gene expression analysis

Gene expression analysis was accomplished by real-time PCR utilizing an ABI 7500 Real Time PCR System (Applied Biosystems/Life Technologies). Total RNA samples (2 *μ*g) were reverse transcribed using the High Capacity cDNA Reverse Transcription Kit (part no. 4368814, Applied Biosystems/Life Technologies) according to the manufacturer’s protocol. Real-time primers and probes for *CEACAM6* (Hs00366002_m1), *CDH1* (Hs00170423_m1), *CST6* (Hs00154599_m1), *ESR1* (Hs00174860_m1), *GNA11* (Hs01588833_m1), *MUC1* (Hs00159357_m1), *MYB* (Hs00920554_m1), *SCNN1A* (Hs00168906_m1), *TFF3* (Hs00173625_m1) and *β-actin* (Hs99999903_m1) were purchased from Applied Biosystems/Life Technologies. All real-time PCR reactions were performed in triplicate using TaqMan Universal PCR Master mix (cat no. 4324018, Applied Biosystems/Life Technologies) in 20 *μ*l volume (10 *μ*l TaqMan Universal PCR Master mix, 1.0 *μ*l TaqMan real-time primers and probes, and 9 *μ*l cDNA and nuclease-free water) and the following amplification conditions: 95°C for 10 min, 40 cycles of 95°C for 15 sec and 60°C for 1 min. Relative expression levels for each gene were calculated based upon the expression of *β-actin* for each sample and differences in gene expression were determined relative to normal breast tissue from reduction mammoplasties for primary tumors using the comparative Ct method described in the ABI Prism 7700 User Bulletin #2 (Applied Biosystems/Life Technologies).

### Statistical analysis

The values for the mean and standard error of the mean (SEM) were calculated using the statistical function of Microsoft Excel 2007. Statistical significance was determined using an unpaired t-test (two-tailed). Error bars depicted in bar graphs represent SEM of 3–6 independent experiments.

## Results

### Breast cancers with aberrant DNA hypermethylation express diminished levels of regulatory miRs

Our previous investigations identified aberrant DNA hypermethylation (characterized by concurrent methylation-dependent gene silencing events) that is significantly associated with the basal-like subtype of breast cancer (12). The aberrant DNA hypermethylation occurs secondary to DNMT hyperactivity and overexpression of DNMT3b (12). In this study, we investigated possible molecular mechanisms governing DNMT3b overexpression driving aberrant DNA hypermethylation, with a focus on miR-mediated regulation of *DNMT3b* in basal-like breast cancers. Hence, we examined the levels of expression of select miRs that are known or predicted to regulate *DNMT3b* (miR-26a, miR-26b, miR-29a, miR-29b, miR-29c, miR-148a, miR-148b, miR-203 and miR-222) among primary breast cancers. We utilized a cohort of 70 primary human breast cancers of known clinical classification representing each of the intrinsic molecular subtypes (36 luminal A-like, 13 luminal B-like, 5 Her2-enriched and 16 basal-like) and 18 normal mammoplasty tissues to analyze expression of microRNAs that contribute to regulation of *DNMT3b*. Average miR expression for breast cancers reflecting each of the clinical classifications is shown in Table I. Among the miRs evaluated, breast cancers associated with specific clinical classifications displayed distinguishing levels of expression. Significantly reduced average expression of miR-29c distinguished basal-like breast cancers from other clinical subtypes (Table I). Likewise, HER2-enriched breast cancers expressed miR-29b at lower levels and the luminal A-like breast cancers expressed miR-203 at lower levels compared to the other breast cancer subtypes (Table I).

**Figure 1.**
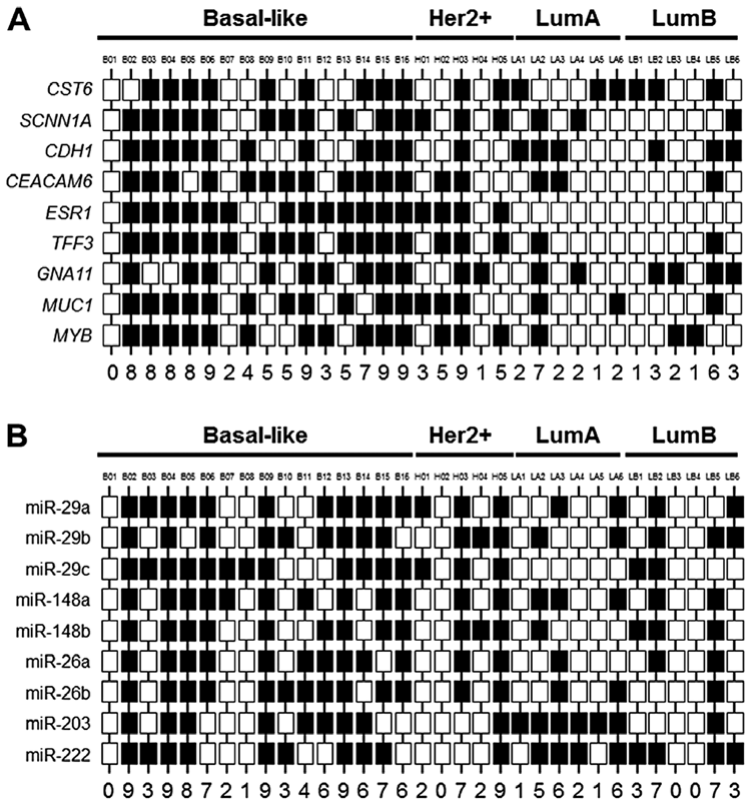
(A) Gene expression patterns of methylation-sensitive genes for primary breast cancers and (B) miR expression patterns and miR scores for primary breast cancers. (A) Black boxes indicate a measured level of expression for an individual gene that is below the median level of expression for the dataset, and white boxes indicate a measured level of expression for an individual gene that is above the median level of expression for the dataset. The numbers at the bottom of each column indicate the gene expression score which reflects the number of methylation-sensitive genes expressed at diminished levels in an individual cancer. (B) Black boxes indicate a level of expression for an individual miR below the median value for the dataset, and white boxes indicate a level of expression of an individual miR that is above the median value for the dataset. The numbers at the bottom of each column indicate the miR score which represents a measure of the number of miRs expressed at diminished levels in an individual breast cancer. Individual breast cancers are designated with: B, basal-like; H, HER2-enriched; LA, luminal A-like; and LB, luminal B-like.

The methylation status of a subset of 33 breast cancers (6 luminal A-like, 6 luminal B-like, 5 HER2-enriched, and 16 basal-like) was established through examination of methylation-sensitive biomarker gene expression. Individual cancers were classified as having aberrant DNA hypermethylation when their expression signature reflected diminished levels of ≥7 epigenetic biomarker genes. Among this cohort of 33 cancers, 11 (33%) were classified as having aberrant DNA hypermethylation (Fig. 1A). A total of 9/11 (82%) breast cancers exhibiting aberrant DNA hypermethylation corresponded to the basal-like subtype, and this group contains 56% (9/16) of all basal-like cancers examined (Fig. 1A). The remaining breast cancers exhibiting aberrant DNA hypermethylation correspond to the luminal A-like (n=1) and HER2-enriched (n=1) subtypes. This finding is consistent with the observation of a large degree of correspondence and overlap between basal-like breast cancers and breast cancers exhibiting aberrant DNA hypermethylation. The miR expression status within this group of 33 breast cancers is shown in Fig. 1B.

Gene expression analysis identified two subsets of basal-like breast cancers - those exhibiting aberrant DNA hypermethylation (n=9, 56%) and those lacking aberrant DNA hypermethylation (n=7, 44%) (Fig. 1A). The basal-like breast cancers exhibiting aberrant DNA hypermethylation include B02–B06, B11 and B14–B16 (Fig. 1A). While there was variability in miR expression among the basal-like breast cancers examined, in general the cancers exhibiting aberrant DNA hypermethylation also expressed diminished levels of regulatory miRs compared to the cancers lacking aberrant DNA hypermethylation (Fig. 1B). However, miR-29c did not display the pattern of expression observed with the majority of regulatory miRs evaluated. Since loss of miR-29c differentiated the basal-like cancers from other subtypes of breast cancers (Table I), the absence of differential expression among the basal-like cancers suggests that loss of miR-29c to be a feature of this clinical breast cancer subtype, irrespective of aberrant DNA hypermethylation status.

**Figure 2.**
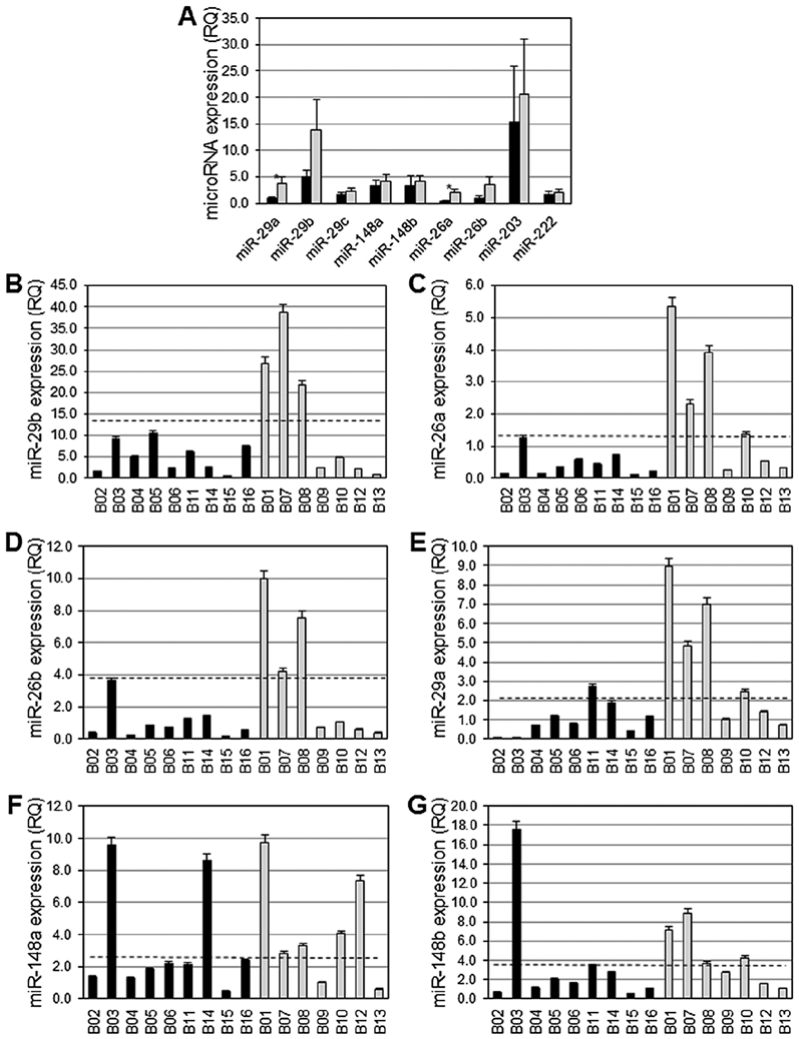
Differential miR expression in basal-like breast cancers with and without aberrant DNA hypermethylation. (A) Black bars represent average miR expression among basal-like breast cancers exhibiting aberrant DNA methylation (n=9), and grey bars represent average miR expression among basal-like breast cancers that lack aberrant DNA hypermethylation (n=7). Comparison of the observed expression levels between groups of basal-like breast cancers was accomplished using an unpaired t-test (two-tailed). The levels of expression for miR-29a and miR-26a were significantly different between basal-like breast cancers that exhibit and lack aberrant DNA hypermethylation (p= 0.03). (B–G) Black bars represent miR expression levels for individual basal-like breast cancers that exhibit aberrant DNA hypermethylation, and grey bars represent miR expression levels for individual basal-like breast cancers that lack aberrant DNA methylation. The black dashed line represents the optimal threshold value determined by Baysian analysis for correct assignments related to aberrant DNA hypermethylation status of individual breast cancers. Each real-time assay was performed in triplicate and the error bars represent SEM. (B) miR-29b, (C) miR-26a, (D) miR-26b, (E) miR-29a, (F) miR-148a, and (G) miR-148b.

The average expression of miR-29a and miR-26a among basal-like breast cancers exhibiting aberrant DNA hyper-methylation was significantly diminished compared to the average expression of these miRs among basal-like breast cancers lacking aberrant DNA hypermethylation (p= 0.03) (Fig. 2A). Differences in the average expression level of miR-29b and miR-26b among basal-like breast cancers exhibiting aberrant DNA hypermethylation versus those lacking aberrant DNA hypermethylation did not reach statistical significance (Fig. 2A), although there was a distinct trend towards lower expression in the basal-like breast with aberrant DNA hypermethylation (p=0.11 and p=0.08, respectively). A total of 9/9 (100%) breast cancers exhibiting aberrant DNA hypermethylation expressed low levels of miR-29b, miR-26a and miR-26b, and 8/9 (89%) of these expressed low levels of miR-29a (Fig. 2B–E). However, among breast cancers lacking aberrant DNA hypermethylation, miR-29a and miR-26a were normally expressed in 4/7 (57%) cancers, and miR-29b and miR-26b were expressed at normal levels in 3/7 (43%) non-hypermethylator cancers (Fig. 2B–E). Interestingly, the three breast cancers lacking aberrant DNA hypermethylation with low levels of expression of miR-29a exhibited low levels of expression of miR-26a, miR-29b and miR-26b. In addition, these three cancers express low levels of miR-148b and miR-203. A total of 7/9 (78%) and 8/9 (89%) basal-like breast cancers exhibiting aberrant DNA hypermethylation had diminished levels of expression of miR-148a and miR-148b, respectively (Fig. 2F–G). These miRs were expressed at normal levels in 5/7 (71%, miR-148a) and 4/7 (57%, miR-148b) basal-like breast cancers that lack aberrant DNA hypermethylation (Fig. 2F–G). A total of 7/9 (78%) breast cancers exhibiting aberrant DNA hypermethylation expressed miR-203 at low levels, while 3/7 (43%) breast cancers lacking aberrant DNA hypermethylation expressed miR-203 at easily detectable levels (data not shown). miR-222 was expressed at low levels in 5/9 (56%) basal-like breast cancers exhibiting aberrant DNA hyper-methylation, while 6/7 (87%) basal-like breast cancers lacking aberrant DNA hypermethylation expressed miR-222 at normal levels (data not shown).

### Diminished expression of miR-29a, miR-29b, miR-26a, miR-26b, miR-148a and miR-148b predict aberrant DNA hyper-methylation among breast cancers

We observed differential expression of miR-29a, miR-29b, miR-26a, miR-26b, miR-148a and miR-148b among basal-like breast cancers with strong trends towards diminished expression in those that exhibiting aberrant DNA hypermethylation compared to those that do not. To assess the value of individual miR expression levels in the prediction of aberrant DNA hypermethylation status of a certain tumor, a Bayesian analysis was performed. Correct assignments (CA) were used as a guiding principle to determine the threshold values for each of the differentially expressed miRs indicated in Fig. 2. The expression level of miR-26a (CA, 81%) emerged as the best individual predictor of aberrant DNA hypermethylation status among basal-like breast cancers, followed by miR-29a (CA, 75%), miR-29b (CA, 75%), miR-26b (CA, 75%), miR-148a (CA, 75%) and miR-148b (CA, 75%) (Table II). These miRs individually displayed excellent sensitivity (range, 78–100%) and negative predictive value (NPV range, 71–100%), as well as good specificity (range, 43–71%) and positive predictive value (PPV range, 69–78%). The remaining miRs displayed poor predictive value for determination of aberrant DNA hypermethylation status among breast cancers (CA, 63–69%) (Table II).

### miR scores correlate with methylation-sensitive gene expression scores among primary breast cancers

miR scores were generated for each basal-like breast cancer, reflecting the number of miRs with diminished expression. miR expression patterns revealed two groups among basal-like breast cancers corresponding to those with low expression and those with high expression (Fig. 1B). Low expression is defined as diminished expression of ≥6 regulatory miRs (n=11 basal-like breast cancers) and high expression is defined as normal expression of ≥3 regulatory miRs (n=5 basal-like breast cancers) (Fig. 1B). Basal-like breast cancers exhibiting aberrant DNA hypermethylation frequently express diminished levels of this panel of miRs. A total of 7/9 (78%) basal-like cancers with aberrant DNA hypermethylation express ≥6 regulatory miRs at diminished levels (Fig. 1B), resulting in higher miR scores. Two basal-like breast cancers exhibiting aberrant DNA hypermethylation expressed diminished levels of all nine miRs examined (Fig. 1B). In contrast to these these basal-like breast cancers, basal-like breast cancers lacking aberrant DNA hypermethylation typically express the majority of these regulatory miRs at higher levels. A total of 4/7 (57%) basal-like breast cancers lacking aberrant DNA hypermethylation cancers express ≥7 miRs at higher levels (Fig. 1B), resulting in lower miR scores. The relationship between aberrant DNA hypermethylation status and miR score is complicated by the observation of two basal-like breast cancers exhibiting aberrant DNA hypermethylation that express most regulator miRs (n=6–7) at normal levels. Likewise, two basal-like breast cancers lacking aberrant DNA hypermethylation express all 9 regulatory miRs at reduced levels. Basal-like breast cancers with aberrant DNA hypermethylation exhibit an average miR score of 6.6±0.7, whereas, basal-like breast cancers lacking aberrant DNA hypermethylation exhibit an average miR score of 4.2±1.4 (NS).

**Figure 3.**
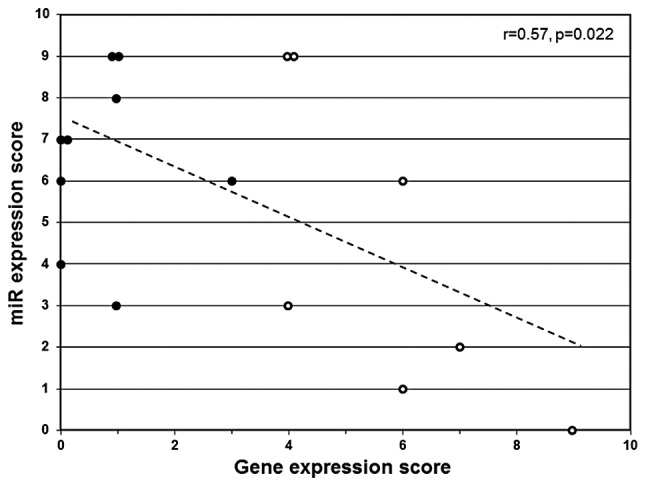
miR expression patterns correlate with promoter methylation status among basal-like breast cancers. Correlation of miR expression patterns (miR expression score) with gene expression levels based on qPCR results (gene expression score) for methylation-sensitive genes among basal-like breast cancers that exhibit or lack aberrant DNA hypermethylation. Closed circles indicate individual basal-like breast cancers with aberrant DNA hypermethylation and open circles indicate individual basal-like breast cancers lacking aberrant DNA hypermethylation.

A linear correlation analysis was performed to determine if miR scores significantly correlate with expression score among basal-like breast cancers. The expression score reflects the combined relative gene expression status for methylation-sensitive biomarker genes associated with aberrant DNA hypermethylation (*CEACAM6*, *CDH1*, *CST6*, *ESR1*, *GNA11*, *MUC1*, *MYB*, *TFF3* and *SCNN1A*) (12). A significant correlation (r= 0.57, p= 0.022) was observed between miR score and gene expression score (Fig. 3). The cancers that exhibit diminished expression of multiple regulatory miRs (high miR score) tend to express low levels of methylation-sensitive genes (gene expression score) and cancers that express higher levels of regulatory miRs (low miR score) tend to express methylation-sensitive genes at higher levels (Fig. 3).

### Co-regulation of miR expression among primary breast cancers

**Figure 4.**
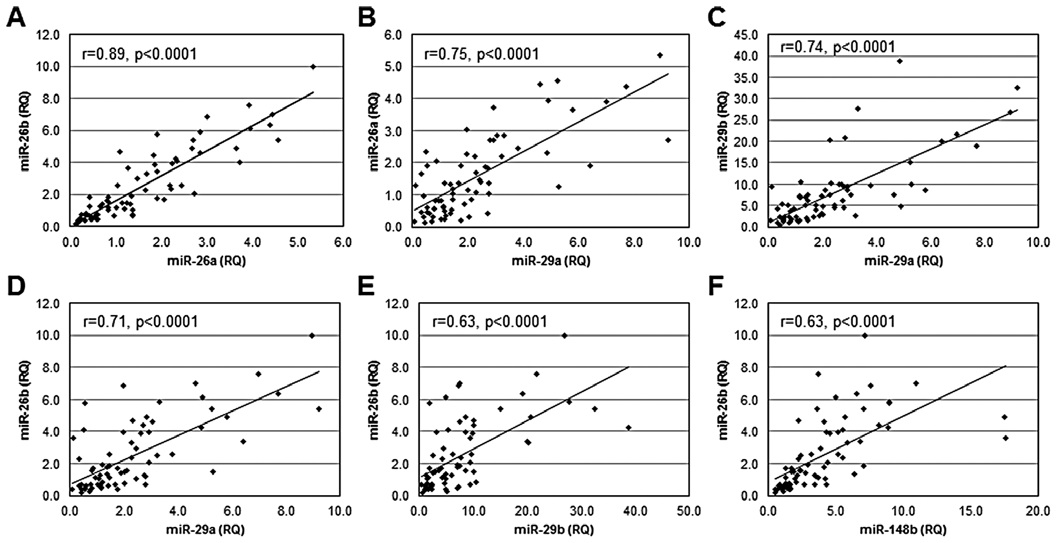
Co-regulation of miR expression among primary breast cancers. Primary breast cancers demonstrate statistically significant relationships between miR expression levels suggesting co-regulation of miRs that regulate *DNMT3b*. The black line represents the linear regression trend line (p-values are indicated). Association of expression between (A) miR-26a and miR-26b; (B) miR-29a and miR-26a; (C) miR-29a and miR-29b; (D) miR-29a and miR-26b; (E) miR-29b and miR-26b; and (F) miR-148b and miR-26b.

To determine if miRs that regulate *DNMT3b* are independently regulated or co-regulated at the level of expression, a linear correlation analysis was performed to examine patterns of miR expression among primary breast cancers. Statistically significant linear relationships were observed between the levels of expression of several miRs (Fig. 4): miR-26a and miR-26b (r=0.89, p<0.0001); miR-29a and miR-26a (r= 0.75, p<0.0001); miR-29a and miR-29b (r=0.74, p<0.0001); miR-29a and miR-26b (r=0.71, p<0.0001); miR-29b and miR-26b (r=0.63, p<0.0001); and miR-148b and miR-26b (r= 0.63, p<0.0001). In addition, significant linear relationships were observed for expression of miR-26a and miR-203 (r=0.71, p=0.0019); miR-26b and miR-203 (r=0.68, p=0.038); miR-26a and miR-29c (r=0.60, p=0.014), miR-148a and miR-203 (r=0.60, p=0.014), and miR-26b and miR-148b (r= 0.50, p= 0.04). No significant linear relationships were observed for expression of miR-26b and miR-29c; miR-148b and miR-203; or miR-29c and miR-203. Combined, these observations suggest that the miRs that function in the regulation of *DNMT3b* are co-regulated.

## Discussion

Epigenetic changes play an important role in normal regulation of gene expression and loss of regulation can significantly contribute to cancer initiation, development and progression (46,47). Epigenetic aberrations such as the silencing of tumor suppressor genes and other negative mediators of neoplastic development have been documented in breast carcinogenesis (11,48,49). The CpG island methylator phenotype (or CIMP) represents a major epigenetic mechanism that has been recognized to contribute significantly to colorectal carcinogenesis as well as to cancers affecting other tissues (50–52). In previous studies, we identified a subset of breast cancer cell lines and primary breast cancers that exhibit aberrant DNA hypermethylation that results in concurrent epigenetic silencing of multiple methylation-sensitive genes (including *CEACAM6*, *CDH1*, *CST6*, *ESR1*, *GNA11*, *MUC1*, *MYB*, *TFF3* and *SCNN1A*) secondary to DNA methyltransferase enzyme hyper activity associated with overexpression of DNMT3b (12). Mining of microarray-based expression data identified the gene expression signature associated with aberrant DNA hyper-methylation in primary sporadic invasive breast cancers (12). A significant correspondence was observed between aberrant DNA hypermethylation and the basal-like molecular subtype of breast cancers (12). Many basal-like breast cancers exhibit the silencing of genes associated with DNMT3b protein over-expression. This observation strongly suggests that the unique features of basal-like breast cancers (poor clinical outcomes, variable response to chemotherapy and recurrence following chemotherapy) may be a direct consequence of methylation-dependent gene silencing events associated with DNMT3b overexpression. This fundamental observation related to the basal-like breast cancers underscores the significance of understanding the mechanism contributing to the overexpression of DNMT3b in these deadly breast cancers.

Several studies have established that miRs exhibit altered expression in cancer tissues compared to normal tissues suggesting that miRs have a role in defining the molecular and pathological profiles of cancers including breast cancer (29,53,54). In addition, miRs have been established as key players in carcinogenesis, with oncogenic or tumor suppressor-like functions (17). Our results suggest loss of combinations of miR-29a, miR-29b, miR-148a, miR-148b, miR-26a and miR-26b is associated with aberrant DNA hypermethylation in primary invasive breast cancers, consistent with the idea that these miRs function as negative mediators of DNMT3b-mediated aberrant DNA hypermethylation. We have also observed a significant concordance between basal-like breast cancers that exhibit aberrant DNA hypermethylation and the group of primary cancers with diminished expression of regulatory miRs. Loss/ reduced levels of these miRs has been documented in various forms of cancer, supporting the suggestion that these miRs possess tumor suppressor-like function. miR-29a and miR-29b are downregulated in chronic lymphocytic leukemia, acute myeloid leukemia, lung cancers, cholangiocarcinoma and prostate cancer (35,55–59). Diminished expression of miR-26a occurs in hepatocellular carcinoma, oral squamous cell carcinoma, bladder cancer, thyroid anaplastic carcinoma, Burkitt’s lymphoma, acute myeloid leukemia, papillary carcinoma, prostate cancer and breast cancer (18,60,61). miR-26b expression is decreased in Hodgkin’s lymphoma, oral squamous cell carcinoma and prostate cancers (61). miR-29c expression is depressed in nasopharyngeal carcinomas, bladder cancers, chronic lymphocytic leukemia, acute myeloid leukemia, lung cancers, cholangiocarcinoma, esophageal squamous cell carcinoma and pancreatic ductal adenocarcinoma (18,55,60–62). miR-148a is downregulated in breast cancers, papillary thyroid carcinoma, pancreatic ductal adenocarcinoma, prostate cancer and colorectal adenocarcinoma (60,61). miR-148b is expressed at diminished levels in oral squamous cell carcinoma, papillary thyroid carcinoma, prostate cancer, colorectal adenocarcinoma and pancreatic ductal adenocarcinoma (61). These studies from the literature provide evidence of loss or diminished expression of these regulatory miRs in various forms of cancer, including breast in some cases.

Multiple mechanisms contribute to miR dysregulation in cancer, including genetic abnormalities (such as chromosomal rearrangement, deletion, amplification or sequence mutations) and epigenetic changes (methylation-dependent silencing of miR expression or alterations in the miRNA biogenesis machinery) (18). More than half of the miR genes (>50%) are located within or in close proximity to chromosomal fragile sites and other genomic regions associated with cancer (18). These sites are prone to genetic alterations and changes in these chromosomal regions result in dramatic alteration of miR expression levels (18). Similarly, numerous studies report promoter hypermethylation as an important mechanism leading to loss of miR expression in cancer (17). miR-148a and miR148b are susceptible to methylation-dependent silencing in cancer (17). These observations suggest that loss of regulatory miR expression leading to *DNMT3b* dysregulation could be the result of genetic or epigenetic mechanisms. Further investigation will be required to establish which of these potential mechanisms contribute to miR dysregulation in basal-like breast cancer.

## Figures and Tables

**Table I.** Average miR expression levels among breast cancers representing clinical subtypes.

miR species	Luminal A-like (n=36)	Luminal B-like (n=13)	HER2-enriched (n=5)	Basal-like (n=16)
miR-29a	2.4±0.3	2.4±0.6	2.0±0.5	2.2±0.6
miR-29b	7.4±1.2	8.0±1.6	4.9±1.5	8.9±2.7
miR-29c	5.7±1.1	8.5±3.4	3.2±0.7	1.9±0.4
miR-148a	3.6±0.5	3.4±0.9	2.8±0.6	3.7±0.8
miR-148b	4.1±0.6	4.3±0.6	3.0±0.7	3.7±1.1
miR-26a	1.8±0.2	1.7±0.3	1.4±0.5	1.1±0.4
miR-26b	2.6±0.3	3.0±0.5	1.6±0.5	2.1±0.7
miR-203	3.7±0.8	11.8±3.3	18.7±9.6	17.7±7.3
miR-222	1.4±0.4	1.3±0.3	2.4±1.0	1.7±0.5

**Table II.** Bayesian analyses show that loss of regulatory miR expression is associated with expression of aberrant DNA hyper-methylation among primary basal-like breast cancers.

miR species	Sensitivity (%)	Specificity (%)	Positive predictive value (%)	Negative predictive value (%)	Correct assignments (%)
miR-29a	89	58	73	80	75
miR-29b	100	43	69	100	75
miR-29c	67	43	60	50	63
miR-148a	78	71	78	71	75
miR-148b	89	57	73	80	75
miR-26a	100	57	75	100	81
miR-26b	100	43	69	100	75
miR-203	78	43	64	60	63
miR-222	56	86	83	60	69
